# Peripheral artery disease in patients with schizophrenia as compared to controls

**DOI:** 10.1186/s12872-023-03143-9

**Published:** 2023-03-08

**Authors:** Linea Rosenberg Jørgensen, Cathrine Linnea Hegtmann, Sune P. V. Straszek, Christian Høyer, Christoffer Polcwiartek, Lars J. Petersen, Martin Kamp Dalgaard, Svend Eggert Jensen, René Ernst Nielsen

**Affiliations:** 1 Psychiatry - Aalborg University Hospital, Sdr. Skovvej 15, 9000 Aalborg, Denmark; 2 Department of Clinical Physiology, Viborg Regional Hospital, Viborg, Denmark; 3 Department of Cardiology, Aalborg University Hospital, Aalborg, Denmark; 4 Department of Nuclear Medicine, Aalborg University Hospital, Aalborg, Denmark; 5 Department of Clinical Medicine, Aalborg University, Aalborg, Denmark; 6 Unit for Psychiatric Research, Psychiatry - Aalborg University Hospital, Aalborg, Denmark

**Keywords:** Atherosclerosis, Toe–brachial index, Cardiovascular disease, Schizophrenia, Peripheral artery disease

## Abstract

**Background:**

Patients with schizophrenia have an increased prevalence of risk factors for peripheral artery disease (PAD) and is expected to have an increased prevalence of PAD. PAD can be detected utilizing toe–brachial index (TBI) which screens for vascular pathology proximal to the toes.

**Methods:**

Using a cross-sectional design, we defined the subpopulations: (1) Patients diagnosed with schizophrenia less than 2 years before inclusion (SCZ < 2), (2) Psychiatric healthy controls matched to subpopulation 1 on sex, age, and smoking status, and (3) Patients diagnosed with schizophrenia 10 or more years before inclusion (SCZ ≥ 10). TBI was calculated by dividing toe pressures by systolic brachial blood pressure, and PAD was defined by TBI < 0.70. Logistic regression analysis with PAD as outcome and sex, age, smoking status, BMI, skin temperature, diagnosis of schizophrenia, and comorbidities as explanatory variables was conducted.

**Results:**

PAD was present in 26.2% of patients diagnosed with SCZ < 2 (17 of 65) and in 18.5% of psychiatric healthy controls (12 of 65) with no statistically significant difference in prevalence rates (*p* = 0.29). PAD was present in 22.0% of patients diagnosed with SCZ ≥ 10 (31 of 141). In logistic regression, patients diagnosed with SCZ < 2 had an increased odds of PAD with psychiatric healthy controls as reference (Odds ratio = 2.80, 95% confidence interval 1.09–7.23, *p* = 0.03). The analysis was adjusted for age, sex, smoking status, BMI and comorbidities such as hypertension, diabetes and heart disease.

**Conclusions:**

This study did not find statistically significant increased prevalence rates of PAD in patients with schizophrenia * even though patients with SCZ were* compared to psychiatric healthy controls using TBI. Utilizing logistic regression PAD was associated with schizophrenia diagnosis within the last 2 years, age and skin temperature. As PAD is initially asymptomatic, screening could be relevant in patients with schizophrenia if other risk factors are prevalent. Further large-scale multicenter studies are warranted to investigate schizophrenia as a potential risk factor for PAD.

*Trial registration*: Clinicaltrials.gov identifier NCT02885792.

## Background

Patients with schizophrenia have a reduced life expectancy by 15–20 years compared to the general population, and mortality rates have been increasing for the last 20 years when compared to the general population [1]. Although patients with schizophrenia have an increased rate of suicide and mortality due to accidents, the most frequent cause of death is cardiovascular disease [2].

Secondary preventive measures have reduced death rates related to cardiovascular disease in the general population by screening patients using risk scores to evaluate the need for preventive measures [3]. For patients with schizophrenia, the PRIMROSE risk score has been developed, enabling risk estimation to initiate primary prophylactic treatment [3, 4]. Despite efforts to minimize mortality rates and improve treatment of cardiovascular risk factors, current practice has not resulted in reduced relative mortality or morbidity rates in patients with schizophrenia [2].

Patients with schizophrenia have more frequent risk factors of cardiovascular diseases such as diabetes, smoking, high levels of LDL-cholesterol, hypertension, and obesity as compared to the general population [6]. Peripheral artery disease (PAD), a cardiovascular disease primarily caused by atherosclerosis, is associated with an overall 10-year all-cause mortality of 56–75% depending on the severity of the disease [5]. A register-based study found an adjusted hazard ratio (HR) of 1.26 for PAD among patients with schizophrenia compared to patients without schizophrenia [7]. The current literature is primarily based on investigations of PAD in patients with symptoms of PAD. The true prevalence of PAD, in a sample not selected due to symptoms, diagnosis or suspicion of atherosclerosis or cardiovascular diseases is not as thoroughly described.

Early diagnosis of PAD in patients with schizophrenia might lead to interventions resulting in lower mortality rates and cardiovascular events as shown in the general population [8]. Toe–brachial index (TBI) screens for occlusive arterial disease proximal to the toes as an index of systolic toe blood pressure over systolic brachial blood pressure [9, 10]. The diagnostic limits of TBI and prevalence rates of PAD has been suggested to be TBI < 0.70/ < 0.64/ < 0.50. TBI < 0.50 is particularly interesting due to the increased risk of cardiovascular and overall mortality in TBI < 0.50 compared to TBI ≥ 0.50 [11, 12].

The aim of the present study was to establish prevalence rates of PAD using TBI < 0.70 among patients with schizophrenia. In supplementary analyses, prevalence rates using TBI < 0.64/ < 0.50 will also be explored [11, 12]. Secondary outcomes included (1) a comparison of TBI between subpopulations of schizophrenia and controls, and (2) an analysis of associations between TBI and known risk factors.

## Methods

### Study design

This was a cross-sectional study based on data from an ongoing clinical prospective cohort study conducted in the North Denmark Region in Denmark (ClinicalTrials.gov identifier: NCT02885792) [13]. The participants in this study were not included on any clinical indication which includes PAD or cardiovascular disease. The present study is therefore able to report results without confounding by indication. The patients were referred to the clinical cohort study from hospital departments both in- and outpatients.

### Study population

The study population comprised three subpopulations; (1) Patients diagnosed with schizophrenia less than 2 years before inclusion in the study (patients diagnosed with SCZ < 2), (2) Psychiatric healthy controls (PHC) with no history of mental illness matched to patients diagnosed with SCZ < 2 on age, sex, and smoking status (smoker/non-smoker) at the time of inclusion, and (3) Patients diagnosed with schizophrenia 10 or more years before inclusion (patients diagnosed with SCZ ≥ 10). Patients with schizophrenia were recruited from the North Denmark Region. Only patients 18 years or older at inclusion diagnosed with schizophrenia or schizo-affective disorder (ICD-10 F20 or F25) and being able to give written informed consent, were included in the study. In Denmark, schizophrenia is defined according to ICD-10, which lists first-rank symptoms, delusions of bizarre characteristics or a combination of at least two of the following symptoms: hallucinations on a daily basis combined with delusions, catatonia, negative symptoms or disorganized thinking.

First-rank symptoms include auditory hallucinations commenting or conversing, somatic hallucinations, thought withdrawal or thought broadcasting. The presence of a brain disorder, drug intoxication or withdrawal hinder the diagnosis of schizophrenia.Patients were excluded if pregnant, breastfeeding or if they were unable to participate in the planned program for the primary cohort study [13].

Using a random recruitment approach, psychiatrically healthy controls were matched on age, sex, and smoking status to patients diagnosed with SCZ < 2. They were invited to participate in this comprehensive cardiac screening programme.

The clinical prospective cohort study has been approved by The North Denmark Region Committee on Health Research Ethics (N-20140047) and is consistent with the ethical standards of the Declaration of Helsinki 2013. The personal data categories collected by the project has been registered in the processing activities of research in the North Denmark Region in compliance with the European Union’s GDPR article 30.

### TBI procedure

Participants rested in a supine position for at least 10 min prior to measurements, and their toes were heated to a temperature above 27 °C using heating overlays prior to testing. Systolic blood pressures were measured in the brachial arteries, and the highest systolic blood pressure measured identified the reference arm (Omron M6^®^ AC, Omron Healthcare Co., Ltd., Kyoto, Japan). The brachial pressure was thereafter measured in the reference arm simultaneously with the measurements of the toe blood pressure. Toe blood pressures were measured with an automated photoplethysmography device (SysToe^®^, Atys Medical, Soucieu-en-Jarrest, France) [14]. Toe pressures were measured consecutively and for each hallux. The measurements of toe pressures were performed until obtaining a difference of 10 mmHg or less between the measured values with a maximum of five measurements of toe pressure for each hallux.

### Definition of TBI and PAD

TBI was calculated using the mean of the systolic toe pressure of the two closest measurements in each hallux and the mean of the two corresponding brachial systolic pressures in the reference arm. The lowest TBI of either extremity was used for the analysis. In case of previous amputation, the TBI from the remaining toe was used for the analysis. If the procedure described above was not possible, the measurements was excluded from further analysis. PAD was defined as TBI < 0.70 with an additional subdivision of prevalence rates of TBI < 0.64 and TBI < 0.50 [11, 12].

### Covariates

The difference in systolic blood pressure between the arms were calculated using the measurements when identifying the reference arm. Skin temperature was measured prior to the measurements of toe pressure for each hallux. Age, sex, smoking status (smoker/non-smoker), body mass index (BMI), and somatic comorbidities were registered in the clinical prospective cohort study [13]. Somatic comorbidities were recorded from electronic medical records, and additional comorbidities diagnosed in other regions or in primary health care were reported by patients or their caregivers.

### Statistical analysis

Mean and standard deviation, median and interquartile range (IQR), or proportions were reported for clinical characteristics, hemodynamic measurements, skin temperature, and BMI. The variables were reported in total for each subpopulation and by TBI above or below 0.70 (TBI ≥ / < 0.70). Continuous variables were compared utilizing a t-test or Mann–Whitney test depending on distribution, and categorial variables were compared utilizing the χ^2^ test for TBI ≥ / < 0.70 for each subpopulation. Hemodynamic variables were compared between subpopulations utilizing Jonckheere-Terpstra statistical test. Plotted values of TBI were presented for each subpopulation with a corresponding boxplot with median, IQR and range.

Differences in prevalence rates were tested using the χ^2^ test for patients diagnosed with SCZ < 2 compared to PHC.

Utilizing logistic regression with PAD as outcome (yes/no), associations with explanatory variables were investigated. Age, sex, smoking status (smoker or non-smoker), skin temperature, BMI, comorbidities, and diagnosis of schizophrenia were utilized as explanatory variables. For patients diagnosed with SCZ < 2, PHC was used as reference. For patients with SCZ ≥ 10, patients diagnosed with SCZ < 2 were used as reference.

*P*-values below 0.05 were considered statistically significant. For statistical analyses, Stata version 16 was used.

## Results

**Fig. 1:**
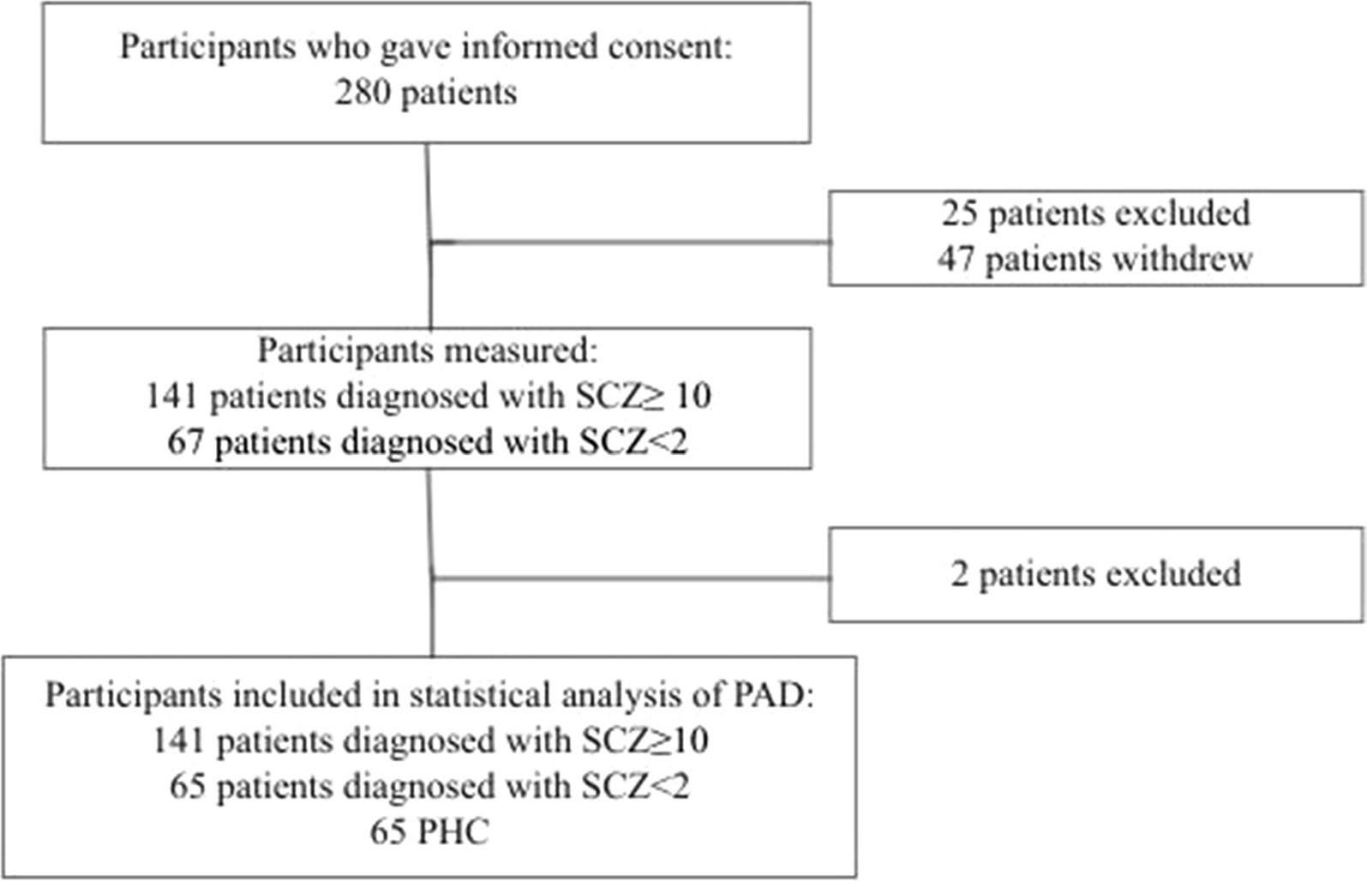
Flowchart of patients included in the study. Patients diagnosed with SCZ < 2 = patients diagnosed with schizophrenia less than 2 years before inclusion. Patients diagnosed with SCZ ≥ 10 = patients diagnosed with schizophrenia ten or more years before inclusion. *PHC* Psychiatric healthy controls. (Title: Fig. 1: Flowchart illustrating the selection process)

In this study, 280 patients with schizophrenia gave consent to be included in the study whereof 25 patients with schizophrenia were excluded as they at baseline had been diagnosed more than 2 years or less than 10 years before inclusion, or not diagnosed with F20 or F25 according to ICD-10. Furthermore, 47 patients with schizophrenia withdrew consent prior to measurement or did not attend planned procedures. Two patients with schizophrenia were excluded from the analyses due to missing blood pressure data. This resulted in a cohort of 65 patients diagnosed with SCZ < 2, and 141 patients diagnosed with SCZ ≥ 10 were included (Fig. 1). 65 PHC were matched to patients diagnosed with SCZ < 2 on sex, age, and smoking status.

### Characteristics of subpopulations

In patients diagnosed with SCZ < 2, the median age was 24.3 years, 38.5% (n = 25) being female. The median age was similar in PHC (median age 24.0). In patients with SCZ ≥ 10, the median age was 49.6 years, 41.8% (n = 59) being female. Results on smoking status, BMI, and comorbidities are shown in Table 1. In the subpopulation of patients diagnosed with SCZ < 2, one patient reported preexisting atherosclerosis in the lower limbs (1.5%) while none reported this condition in the other two subpopulations. The abovementioned patient’s TBI was above 0.70. Regarding all the participants, 4.1% (n = 11) reported heart disease, including tachycardia, heart failure, pacemaker, and sinus node dysfunction, and 1.5% (n = 4) reported dyslipidemia, including hypercholesterolemia and hypertriglyceridemia.

**Table 1:** Patient characteristics stratified by TBI above or below 0.70

Proportion (in %)	Patients diagnosed with SCZ < 2			*p*-value	Psychiatric healthy controls			*p*-value	Patients diagnosed with SCZ ≥ 10			*p*-value
	Total n = 65	TBI < 0.70 n = 17 26.2%	TBI ≥ 0.70 n = 48 73.8%		Total n = 65	TBI < 0.70 n = 12 18.5%	TBI ≥ 0.70 n = 53 81.5%		Total n = 141	TBI < 0.70 n = 31 22.0%	TBI ≥ 0.70 n = 110 78.0%	
Age (median and IQR)	24.3 (21.5–29.4)	25.2 (22.9–27.2)	24.2 (21.5–29.8)	0.91	24.0 (21.1–28.3)	25.9 (22.0–30.6)	23.5 (21.1–28.1)	0.38	49.6 (43.0–56.5)	52.8 (45.2–60.9)	49.3 (42.0–55.4)	0.15
Sex (female)	25(38.5%)	5(29.4%)	20(41.7%)	0.37	25 (38.5%)	2 (16.7%)	23 (43.4%)	0.09	59 (41.8%)	9 (29.0%)	50 (45.5%)	0.10
Smoker	41 (63.1%)	12 (70.6%)	29 (60.4%)	0.46	34 (52.3%)	6 (50.0%)	28 (52.8%)	0.86	62 (44.0%)	14 (45.2%)	48 (43.6%)	0.88
BMI (median and IQR)	28.4 (24.6–33.0)	28.8 (24.9–30.3)	28.0 (23.9–33.8)	0.82	23.7 (21.8–25.8)	24.3 (22.1–26.5)	23.5 (21.8–25.2)	0.48	28.8 (24.7–33.0)	27.3 (23.0–29.6)	29.0 (25.3–33.3)	0.05
Diabetes	4 (6.2%)	1 (5.9%)	3 (6.3%)	0.96	1 (1.5%)	0	1 (1.9%)	0.63	16 (11.4%)	3 (9.7%)	13 (11.8%)	0.74
Hypertension	1 (1.5%)	0	1 (2.1%)	0.55	0	0	0	–	12 (8.5%)	4 (12.9%)	8 (7.3%)	0.32
Heart disease	0	0	0	–	1 (1.5%)	0	1 (1.9%)	0.63	10 (7.1%)	1 (1.7%)	9 (8.2%)	0.34
Dyslipidemia	0	0	0	–	1 (1.5%)	0	1 (1.9%)	0.63	3 (2.1%)	1 (3.2%)	2 (1.8%)	0.63

TBI = toe–brachial index. IQR = interquartile range. BMI = body mass index

P-values are reported for subgroups by TBI above or below 0.70. Statistical test performed are either χ^2^ or Mann–Whitney test. Patients diagnosed with SCZ < 2 = patients diagnosed with schizophrenia less than two years before inclusion. Patients diagnosed with SCZ ≥ 10 = patients diagnosed with schizophrenia 10 or more years before inclusion

Of the patients reported as smokers (n = 137), 121 reported to smoke daily (38 patients diagnosed with SCZ < 2, 23 PHC, and 60 patients diagnosed with SCZ ≥ 10). The median of daily cigarette use of the patients smoking daily was 15 (IQR 10–20, n = 55). Of the non-smokers, 61 reported to be a former smoker (8 patients diagnosed with SCZ < 2, 5 PHC, and 48 patients diagnosed with SCZ ≥ 10).

Sex, age, smoking status, and comorbidities did not differ between TBI ≥ / < 0.70 in each subpopulation. BMI differed when grouped by TBI ≥ / < 0.70 in patients diagnosed with SCZ ≥ 10 (*p* < 0.05).

Sex, age, smoking status, and comorbidities did not differ between patients diagnosed with SCZ < 2 and PHC in total and in TBI ≥ / < 0.70 (*p* all ≥ 0.17). BMI differed between patients diagnosed with SCZ < 2 and PHC in total and in TBI ≥ / < 0.70 (*p* all < 0.001).

Mean toe temperatures during measurement were 33.4 (IQR: 30.9–34.9) for patients diagnosed with SCZ < 2, 30.0 (IQR: 29.0–31.2) for PHC, and 33.3 (IQR: 31.1–35.2) for SCZ ≥ 10 with no significant difference between TBI ≥ / < 0.70 (*p* all ≥ 0.14) in each subpopulation.

### TBI and hemodynamic variables

**Fig. 2:**
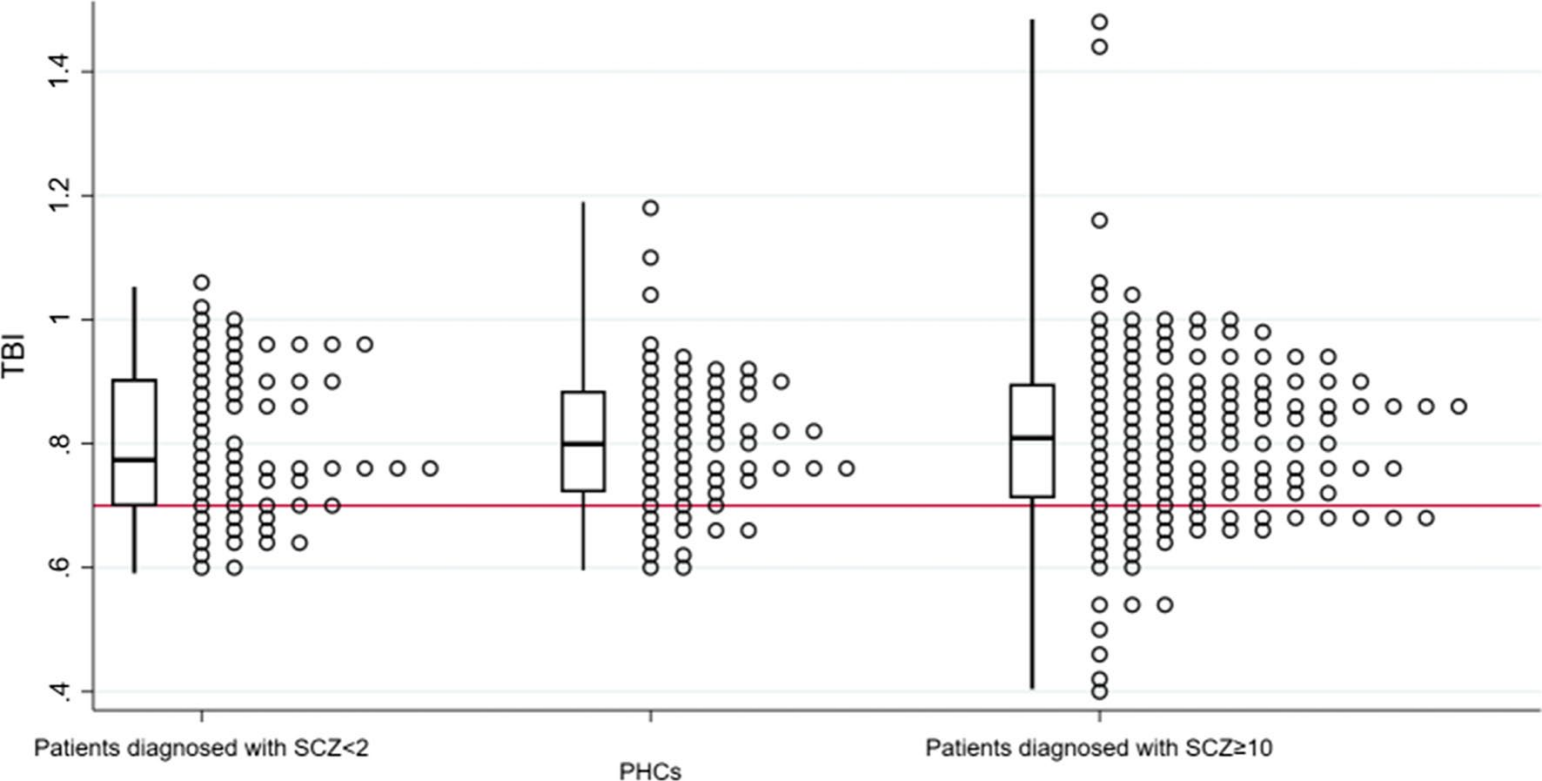
Plotted values of TBI for each subpopulation are presented utilizing a boxplot presenting range and quartiles. The horizontal reference line indicates TBI = 0.70. *TBI* toe–brachial index. *PHC* psychiatric healthy controls. Patients diagnosed with SCZ < 2 = patients diagnosed with schizophrenia less than 2 years before inclusion. Patients diagnosed with SCZ ≥ 10 = patients diagnosed with schizophrenia ten or more years before inclusion. (Title: Fig. 2: Plotted values of TBI for each subpopulation)

Plotted values of TBI for each subpopulation in Fig. 2 and results on TBI, toe pressure, systolic brachial blood pressure and difference in blood pressure between arms in Table 2 show similar median TBI in patients diagnosed with SCZ < 2 as compared to PHC. TBI, systolic toe pressure, systolic brachial blood pressure, and difference in blood pressure between arms did not differ statistically significant between subpopulations (*p* all ≥ 0.15).

**Table 2:** Hemodynamic variables in total or stratified by TBI above or below 0.70

	Patients diagnosed with SCZ < 2			Psychiatric healthy controls			Patients diagnosed with SCZ ≥ 10		
	Total n = 65	TBI < 0.70 n = 17	TBI ≥ 0.70 n = 48	Total n = 65	TBI < 0.70 n = 12	TBI ≥ 0.70 n = 53	Total n = 141	TBI < 0.70 n = 31	TBI ≥ 0.70 n = 110
TBI (median and IQR)	0.77 (0.70–0.90)	0.66 (0.65–0.69)	0.86 (0.76–0.95)	0.80 (0.72–0.88)	0.66 (0.62–0.68)	0.82 (0.76–0.90)	0.81 (0.71–0.90)	0.66 (0.60–0.68)	0.85 (0.77–0.93)
Systolic TP (median and IQR)	98 (88–109)	85 (77–88)	102 (94–120)	104 (91–121)	90 (78–92)	101 (94–113)	98 (89–111)	87 (76–92)	110 (99–127)
Systolic brachial pressure (median and IQR)	119 (108–129)	122 (116–128)	115 (108–129)	120 (111–126)	124 (121–128)	117 (110–126)	125 (116–132)	129 (120–134)	123 (115–131)
Difference in systolic BP between arms (median and IQR)	5 (2–10)	6 (3–9)	5 (2–10)	4 (1–7)	6 (2–8)	3 (1–7)	5 (2–8)	6 (3–10)	4 (2–8)

*TBI* Toe–brachial index, *TP* Toe pressure, *IQR* Interquartile range, *BP* blood pressure

Patients diagnosed with SCZ < 2 = patients diagnosed with schizophrenia less than 2 years before inclusion. Patients diagnosed with SCZ ≥ 10 = patients diagnosed with schizophrenia 10 or more years before inclusion

Systolic brachial blood pressure and difference in systolic blood pressure between arms did not differ statistically significantly between TBI ≥ / < 0.70 in each subpopulation (*p* all >  = 0.06).

### Prevalence of PAD

PAD (defined as TBI < 0.70) was present in 26.2% of the patients diagnosed with SCZ < 2 (n = 17) and in 18.5% of PHC (n = 12) with no statistically significant difference (*p* = 0.29). PAD was present in 22.0% of the patients diagnosed with SCZ ≥ 10 (n = 31). TBI < 0.64 was present in 6.2% of the patients diagnosed with SCZ < 2 (n = 4), in 7.7% of the PHC (n = 5), and in 9.9% of the patients diagnosed with SCZ ≥ 10 (n = 14). TBI < 0.50 was present in patients diagnosed with SCZ ≥ 10 (2.8%, n = 4).

### Associations between explanatory variables and PAD

Logistic regression analysis with PAD (TBI < 0.70) as outcome and sex, age, smoking status, BMI, skin temperature, diagnosis of schizophrenia, and comorbidities as explanatory variables was conducted (Table 3). Sex, smoking status, BMI, patients diagnosed with SCZ ≥ 10 (reference: patients diagnosed with SCZ < 2), diabetes, hypertension or heart disease was not associated with the diagnosis of PAD using logistic regression. Age was slightly associated with PAD (OR 1.04, 95% CI 1.00–1.09, *p*-value = 0.04)—Patients diagnosed with SCZ < 2 was associated with PAD compared to PHC (OR 2.80, 95% CI 1.09–7.23, *p*-value = 0.03). Skin temperature was also associated with PAD (OR 0,88, 95% CI 0.77–1.00, *p*-value = 0.05).

**Table 3:** Multivariable logistic regression of the association between PAD, as defined by TBI < 0.70, and baseline characteristics

	Odds ratio (95% CI)	*p*-value
Sex (man)	1.72 (0.58–5.08)	0.33
Age (years)	1.04 (1.00–1.09)	**0.04**
Smoking status (current smoker)	0.97 (0.52–1.81)	0.93
BMI	1.00 (0.88–1.13)	0.99
Skin temperature of measured foot (degree Celsius)	0.88 (0.77–1.00)	**0.05**
Patients diagnosed with SCZ < 2	2.80 (1.09–7.23)	**0.03**
Patients diagnosed with SCZ ≥ 10	0.30 (0.09–1.04)	0.06
Diabetes	0.85 (0.23–3.12)	0.81
Hypertension	1.54 (0.31–7.69)	0.60
Heart disease	0.37 (0.04–3.36)	0.38

*CI* Confidence interval, *BMI* Body mass index. Bold text indicates statistical significance

Logistic regression of association between TBI < 0.70 (PAD) and baseline characteristics. Psychiatrically healthy controls were used as reference for patients diagnosed with schizophrenia less than 2 years before inclusion. Patients diagnosed with schizophrenia less than 2 years before inclusion were used as reference for patients diagnosed with schizophrenia 10 or more years before inclusion. Patients diagnosed with SCZ < 2 = patients diagnosed with schizophrenia less than 2 years before inclusion. Patients diagnosed with SCZ ≥ 10 = patients diagnosed with schizophrenia 10 or more years before inclusion

## Discussion

PAD was associated with schizophrenia compared to PHC, although prevalence rates were overall similar between patients diagnosed with SCZ < 2, PHC, and patients diagnosed with SCZ ≥ 10. In addition, no difference in the odds ratio of being diagnosed with PAD was observed among patients with SCZ < 2 and SCZ ≥ 10.

### Prevalence rates of PAD and current literature

The prevalence rates of PAD among patients with schizophrenia in this study were higher compared to the rates in current registry-based studies. In patients with schizophrenia and incident acute myocardial infarction, 6.4% had a comorbid diagnosis of PAD (5.8% in the control group), and 11.7% of patients with schizophrenia who died of cardiovascular diseases had a diagnosis of peripheral vascular disease (20.1% among patients without severe mental illness) [15, 16]. This discrepancy could be explained by an underestimation of PAD among patients with schizophrenia, for instance due to fewer diagnostic procedures. However, it should be considered that the current diagnostic limit of TBI < 0.70 could overestimate the prevalence of PAD in this study population. When using TBI < 0.64/ < 0.50, the prevalence rates of PAD were more consistent with the current register-based studies in study populations with known atherosclerosis. However, most of the current study population did not have known atherosclerosis, and therefore the prevalence of PAD brought further evidence of an increased risk of undetected atherosclerosis among patients with schizophrenia. This was in line with other studies suggesting increased risk of undetected myocardial infarction in this patient group compared with the general population [17]. Vetter et al. found peripheral microvascular dysfunction in patients with schizophrenia [18]. Furthermore, Ünsal et al. showed that patients with schizophrenia had a lower ankle-brachial index (ABI) compared to a control group [19]. However, Ünsal et al. found that none of the participants had PAD, as defined by ABI < 0.90. This difference in the prevalence of PAD could be explained by the exclusion of patients with schizophrenia having comorbidities such as diabetes, hypertension, or coronary artery disease, contrary to our study in which patients were not excluded due to comorbidities. Furthermore, ABI have been considered to underestimate the diagnosis of PAD which could also explain the difference between the prevalence rate found in Ünsal et al. [19] compared to this study [19, 20].

### Associations of PAD and risk factors

The associations of PAD were investigated by logistic regression which showed that increasing age resulted in an increase in odds of PAD (OR = 1.04, 95% confidence interval: 1.00–1.09, *p* = 0.04), while sex, smoking status, comorbidities, and BMI did not. The odds ratio was lower with increasing skin temperature (OR = 0.88, *p* = 0.05), indicating that the skin temperature affected the measurement and therefore the diagnosis of PAD. It could also be caused by poorer peripheral blood flow. The non-invasive method infrared thermography visualises the skins temperature in different areas and quantifies any difference in temperature between extremities. This method could have further explored the association between skin temperature and the diagnosis of PAD based on TBI [21].

The most substantial difference in odds ratio was in patients diagnosed with SCZ < 2 as compared to PHC with an increased OR of 2.80 (*p* < 0.05). This finding suggests that there were non-determined factors affecting risk of PAD between patients and controls, which were not adjusted for in the current analysis. Patients diagnosed with SCZ < 2 in the present study were matched to a control group by age, sex, and smoking status, but not on other known risk factors of atherosclerosis. However, no major differences in characteristics between patients diagnosed with SCZ < 2 and PHC were shown when stratified by TBI < 0.70, except for BMI. This suggest that the increased OR among patients diagnosed with SCZ < 2 were not solely due to conventional risk factors when compared to the PHC. This is also termed the cluster of having schizophrenia which describes a combination of early onset of risk factors (smoking, unhealth diet, lack of exercise, obesity), a possible genetic predisposition to cardiovascular disease and schizophrenia, treatment with antipsychotic drugs which increases the risk of body weight gain, dyslipidemia and diabetes and small effects when using lifestyle interventions [2].

Even though the prevalence of atherosclerosis in general increases with age, the OR was highest in the youngest subpopulation with schizophrenia. This is consistent with Hsu et al. who found that the adjusted hazard ratio for PAD among patients with schizophrenia is 1.26-fold higher compared to a cohort of participants without schizophrenia [7]. The highest adjusted HR was increased 1.72-fold which was among patients with schizophrenia aged 20–34 as compared to a cohort of participants not diagnosed with schizophrenia in the same age group [7]. Along with this current study, this suggests that patients with schizophrenia are at risk of PAD at a younger age than the general population. The lower OR in patients diagnosed with SCZ ≥ 10 could be a result of survival bias or changes in lifestyle due to societal development resulting in a higher odds among patients diagnosed with SCZ < 2, who were younger compared to patients diagnosed with SCZ ≥ 10.

### Strengths and limitations of the study

Similar to other studies in this patient group, nearly one-third of the initial cohort of patient with schizophrenia were excluded or withdrew in the present study prior to TBI assessment. Thus, the true prevalence rate was not estimated, but instead the prevalence rates have not been affected by bias caused by clinical indication of PAD or screening of high-risk patients.

In this study, the small difference in smoking status between patients diagnosed with SCZ < 2 and PHC was due to participants stating smoking status differently from time of inclusion to filling out the questionnaire. Information on smoking prior to the measurements of toe pressures could have been useful as smoking results in a contemporary peripheral vasoconstriction which could lead to lower measurements of toe pressures [22].

The patients diagnosed with SCZ ≥ 10 did not have a matched control group which is a major limitation in this study. Therefore, this study should be replicated in a larger setting using a matched control group for patients diagnosed with SCZ ≥ 10.

The chosen diagnostic limit of TBI could result in an overestimation as the prevalence rate was high in PHC who did not have notable known cardiovascular risk factors. At present, the diagnostic usage of TBI is not consistent, and different diagnostic limits have been suggested as well as using TBI as a screening tool [23]. Depending on methods of measuring TBI, heating of toes, cuff size, pretest rest, and/or ambient temperature, diagnostic limits vary from TBI ≤ 0.54 to < 0.75 [11, 23]. The method of measuring TBI in this study was validated in regard to heating of toes, pretest rest, and the automated photoplethysmography device. Only regarding the uncontrolled room temperature, the method of measuring TBI differed [14]. In general, TBI has exhibited better sensitivity than the currently used ABI with fewer disadvantages among patients with medial arterial calcification (MAC), which have primarily been present in elderly people and patients with diabetes [24]. Therefore, TBI could be preferable as a screening tool, and the diagnostic limits should be further validated [11, 24].

Wickström et al. found TBI < 0.50 to be predictive of cardiovascular and overall mortality in patients with symptomatic PAD [12]. In this current study, some of the patients diagnosed with SCZ ≥ 10 had TBI < 0.50. As the patients in this study did not state symptoms, TBI < 0.50 could be predictive of cardiovascular and overall mortality, however not in the same manner as in symptomatic PAD [5, 25]. This study has highlighted the need to establish the diagnostic limit of TBI in regard to increased mortality rates in low-risk populations. This could contribute to specific guidelines on screening and preventive treatment of atherosclerosis among patients with schizophrenia using TBI as a non-invasive screening tool and thereby reduce the relative mortality rate caused by cardiovascular diseases.

## Conclusions

This study did not find statistically significant increased prevalence rates of PAD in patients with schizophrenia even though patients with SCZ were compared to PHC using TBI. The odds ratio of PAD was significant increased in a cohort of patients with SCZ compared to PHC using logistic regression which demonstrates an association. As PAD is initially asymptomatic, screening could be relevant in patients with schizophrenia if other risk factors are prevalent. Further large-scale multicenter studies are warranted to investigate schizophrenia as a potential risk factor for PAD.

## Data Availability

The datasets generated and analyzed during the current study are not publicly available as it includes sensitive data which could identify participants.

## Abbreviations

ABI: Ankle–brachial index
BMI: Body mass index
CI: Confidence interval
GDPR: General Data Protection Regulation
HR: Hazard ratio
ICD-10: International Classification of Diseases version 10
IQR: Interquartile range
MAC: Medial arterial calcification
OR: Odds ratio
PAD: Peripheral artery disease
PHC: Psychiatric healthy controls
SCZ: Schizophrenia
TBI: Toe–brachial index
TP: Toe pressure
